# Toll-like receptor 2 (TLR2) plays a role in controlling cutaneous leishmaniasis in vivo, but does not require activation by parasite lipophosphoglycan

**DOI:** 10.1186/s13071-016-1807-8

**Published:** 2016-10-06

**Authors:** Alice Halliday, Paul A. Bates, Michael L. Chance, Mark J. Taylor

**Affiliations:** 1 Department of Parasitology, Liverpool School of Tropical Medicine, Pembroke Place, Liverpool, L3 5QA UK; 2 Division of Biomedical and Life Sciences, Faculty of Health and Medicine, Furness Building, Lancaster University, Bailrigg, Lancaster LA1 4YG UK

**Keywords:** *Leishmania major*, *Leishmania mexicana*, TLR2, Lipophosphoglycan

## Abstract

**Background:**

Leishmaniasis is a neglected tropical disease affecting millions of individuals worldwide. Despite several studies reporting involvement of the innate immune receptor Toll-like receptor 2 (TLR2) in the recognition of surface glycolipids from *Leishmania* parasites in vitro, the role of TLR2 and its co-receptors during cutaneous leishmaniasis infection in vivo is unknown.

**Methods:**

To explore the role of TLR2 and its co-receptors in cutaneous leishmaniasis, mice deficient in either TLR2, 4, 1 or 6, or wild-type (WT) controls, were infected with either *Leishmania major* promastigotes, *L. mexicana* promastigotes, *L. mexicana* amastigotes, or *LPG1* ^*−/−*^ *L. mexicana* promastigotes. For each infection, lesion sizes were monitored and parasite burden was assessed at various time points. To assess immune responses, draining lymph node (DLN) cells were re-stimulated with parasite antigens and the production of cytokines and parasite-specific antibody isotypes in blood was determined by ELISA.

**Results:**

Mice deficient in TLR2 and TLR4 presented with larger lesions and higher parasite burdens than WT controls. Mice lacking TLR2 co-receptors TLR1 or TLR6 did not show exacerbated infection, suggesting that TLR2 does not require either co-receptor in the recognition of *Leishmania* infection. Furthermore, it appears that lipophosphoglycan (LPG) is not the major mediator of TLR2 activation during infection with *L. mexicana*, as parasites lacking LPG (axenic amastigotes and *LPG1* ^−/−^ promastigotes) also resulted in exacerbated disease in TLR2^−/−^ mice. Infected TLR2^−/−^ mice show a skewed Th2 immune response to *Leishmania* parasites, as demonstrated by elevated IL-4, IL-13 and IL-10 production by DLN cells from *L. mexicana* infected mice in response to antigen. Furthermore, *L. major* infected TLR2^−/−^ mice have elevated antigen-specific IgG1 antibodies.

**Conclusions:**

TLR2 deficiency leads to exacerbation of disease and parasite burden through promotion of Th2 immunity. TLR2 activation in vivo occurs independently of parasite LPG, suggesting other parasite ligands are involved in TLR2 recognition of *Leishmania*.

**Electronic supplementary material:**

The online version of this article (doi:10.1186/s13071-016-1807-8) contains supplementary material, which is available to authorized users.

## Background

Leishmaniasis is a neglected tropical disease (NTD), currently affecting at least 12 million people, with 350 million at risk in 98 countries across the globe [1, 2]. Protozoan parasites from the genus *Leishmania* are the causative agents for leishmaniasis, which encompasses a spectrum of disease types that affect both humans and other animal species. The cutaneous form of leishmaniasis is the most prevalent form of the disease, caused by several different species, two of the most important being *L. major* in the Middle East and North Africa and *L. mexicana* in Central and South America.

The mouse model of *L. major* infection in mice (particularly BALB/c and C57BL/6 strains) has been extensively studied for markers of resistance and susceptibility, and has given useful insight into the type of immune response required for disease control. In particular, the adaptive immune response has been comprehensively examined in C57BL/6 and BALB/c mice infected with *L. major* [3]. For clearance and protection, a robust T helper 1 (Th1) response is required, characterised by production of the cytokine IFNγ, leading to classical activation of macrophages, production of the cytokines TNFα and nitric oxide (NO), and intracellular killing of parasites [3–8]. A more limited number of in vivo studies exploring the role of innate immune recognition of infection on the development of adaptive immunity have also been reported. These studies have identified a role for TLR pathways, as mice lacking the adaptor molecule MyD88, common to most TLRs and IL-1R, were highly susceptible to *L. major* and mounted a non-protective Th2 response [9–11]. A role for TLR4 in controlling *L. major* infection in vivo has been reported [12, 13], but was not reproduced in another study [14], and TLR9 has been shown to play a role in controlling *L. major* infection in vivo [15]. TLR2 has been implicated in the recognition of *Leishmania* parasites in vitro, in particular via sensing of lipophosphoglycan (LPG), the major surface glycolipid present on the infective promastigote stage [10]. It has been reported that activation of TLR2 by LPG results in both a pro-inflammatory phenotype as shown by increased Th1 cytokine production by NK cells [16] and NO production in macrophages [17], but also a regulatory phenotype as shown by increased expression of suppressors of cytokine signalling (SOCS) molecules SOCS-1 and SOCS-3 in murine macrophages [10]. Furthermore, different forms of LPG (i.e. soluble or membrane bound) have been shown to stimulate macrophages to different extents [18].

In this study, mice lacking TLR2, TLR1, TLR6 and TLR4, were infected with *L. major* or *L. mexicana* to determine the role of TLR2 and its known co-receptors in cutaneous leishmaniasis in vivo, and to compare these to TLR4, which has previously been reported to facilitate the control of *L. major* infection.

## Methods

### Parasites and antigens

*Leishmania major* FV1 (MHOM/IL/80/Friedlin; clone V1), *L. mexicana* (MNYC/BZ/62/M379) and the genetically modified *L. mexicana LPG1* ^−/−^ (also M379) were used in this study. Promastigote parasites were cultured in Medium 199 (M199; Invitrogen) containing 10–20 % heat-inactivated foetal bovine serum (HI-FBS) “gold” (PAA), 25 μg/ml gentamicin sulphate and 1× BME vitamins (Sigma), at 26 °C. Axenic amastigotes (of *L. mexicana* parasites only) were cultured in Grace’s medium, supplemented as above and adjusted to pH 5.5, at 32 °C. In the case of both promastigotes and amastigotes, parasites were kept in volumes of 5–55 ml and were sub-passaged at a ratio of 1:2–1:20 in fresh medium every 5–10 days according to growth rate (typically 1:10 every 7 days). Infectivity of parasites was maintained by regular passage of parasites through a susceptible animal.

Freeze-thaw antigen (FTAg) was made from cultured promastigotes as described and developed elsewhere [19, 20]. Stationary-phase promastigotes were washed three times in DPBS and re-suspended at a concentration of 10^9^/ml, and were then subjected to five rapid freezing and thawing cycles at -80 °C and 37 °C, respectively. Protein concentration was measured using the BCA assay and aliquots of FTAg were kept at -80 °C until use. For *L. mexicana* parasites only, washed membrane antigen (WMAg) was generated from cultured axenic amastigotes using hypotonic lysis as described by Thomas et al. [20]. Axenic amastigotes were washed three times in PBS and counted using a haemocytometer before lysis in nuclease-free water containing 0.1 mM TLCK and 1 μg/ml leupeptin at 10^9^ parasites/ml for 5 min on ice. The lysed parasites were then frozen at -80 °C after addition of an equal volume of 0.1 mM TLCK, 1 μg/ml leupeptin, 20 % glycerol. After freezing, the lysed parasites were thawed and centrifuged at 6100× *g* for 10 min (4 °C) to remove PBS containing soluble protein and protease inhibitors before re-suspending membranes at 10^9^/ml in PBS. The WMAg solution was assayed for protein concentration using the BCA assay and aliquots were kept at -80 °C until use.

### Mice and infections

All procedures involving the use of laboratory animals were approved by the Ethics and Animal Care Committees of the University of Liverpool and Liverpool School of Tropical Medicine, and were carried out according to the Animals (Scientific Procedures) Act (UK Home Office), under licenses 40/3514 and 40/2958. WT C57BL/6 mice were purchased from Charles River (UK), while TLR2^−/−^, TLR1^−/−^, TLR6^−/−^ and TLR4^−/−^ mice were originally obtained from Professor Akira (Osaka University, Japan), and have since been maintained at the University of Liverpool. All procedures involving live animals were performed at the BSU in the Duncan Building, University of Liverpool. Female age-matched WT, TLR2^−/−^, TLR1^−/−^, TLR6^−/−^ and TLR4^−/−^ mice were infected with either 10^5^ *L. major* FV1, *L. mexicana* M379 or *L. mexicana* M379 *LPG1* ^−/−^ stationary-phase promastigotes, or *L. mexicana* M379 amastigotes by subcutaneous injection to the shaven rump in a 100 μl volume of HBSS. All parasite cultures were confirmed to be negative for mycoplasma contamination prior to infection. Lesion progression was monitored by taking weekly measurements of lesion size using a metric dial calliper and calculating the overall lesion area (mm^3^) for each animal, and these measurements were used to generate area under the curve (AUC) values. At the end of infection experiments, mice were euthanized and blood was collected via cardiac puncture to allow for the collection of plasma samples. The lesion was removed and either processed for limiting dilution or homogenised for DNA extraction, and the spleens and draining lymph nodes (DLNs) were removed under sterile conditions and processed for cell stimulation experiments.

### Parasite burden quantification, and parasite genotyping

DNA was extracted from the lesion tissue using the DNA Blood and Tissue Kit (Qiagen, Hilden, Germany) according to the manufacturer’s instructions. This method was first validated against the more widely used method of limiting dilution and found to be more sensitive. For quantification of parasites in lesion tissue, a qPCR method was developed based on that described by Nicolas et al. [21] with modifications. The following components were used in each 20 μl reaction: 1× SybrGreen Mastermix (Qiagen), 500 nM of JW11 and JW12 primers, nuclease-free water and 2 μl DNA (samples had concentration between 35 and 150 ng/μl), to amplify a 120 bp region of kinetoplastid DNA. Reactions were performed in duplicate for each sample, in wells of a 96-well high profile white PCR plate (Starlab, Hamburg, Germany). The reaction conditions were as follows: 95 °C for 15 min, followed by 40 amplification cycles at 95 °C for 15 s, 60 °C for 15 s and 72 °C for 15 s. The Chromo 4™ System for real-time PCR detection (BioRad) was used and data were collected using MJ Opticon Monitor Analysis Software Version 3.1 (BioRad). A melting curve was then generated by increasing the temperature from 50 to 95 °C and reading the plate at each 1° increment. A standard curve was included on each plate, where 8 × 10-fold serial dilutions of DNA from cultured *L. major* or *L. mexicana* parasites were diluted in nuclease-free water and spiked with DNA from naive mouse tissue. The following controls were included on each plate in duplicate: no template control (NTC), nuclease-free water, DNA from *Leishmania*-positive lesion, and DNA from naive mouse tissue. Average parasite numbers for reactions were used to estimate total parasite burdens per lesion, by adjusting for total DNA volume from the initial DNA extraction.

To determine the species of *Leishmania* used in each experiment, DNA extracted from lesions was used to amplify an RPS7 intergenic sequence from the genome by PCR, as described in [22]. Briefly, the primer pairs AM1 (5′-CGC GTG TCG TTC GGC TTT ATG TG-3′) and AM2 (5′-CTT ACG GAG CTT GCT GAG GTG AGG-3′) were used to amplify the target region, followed by digestion with restriction enzyme *Msp*I. The pattern of bands formed differs between species: two bands of different sizes between the range of 300–350 bp indicates *L. mexicana*, whilst 2 bands of approximately 500 and 300 bp indicates *L. major*.

### Cell stimulations and immunological techniques

DLN cells and splenocytes were obtained by homogenising DLN and spleen tissue (removed using sterile techniques) using a 70 μm cell strainer (BD) and collecting into Dulbecco’s Modified Essential Medium (DMEM, Invitrogen Carlsbad, CA, USA) medium containing 10× HI-FBS “gold” (PAA), 50 U/ml penicillin and 50 μg/ml streptomycin (Invitrogen). Cells were cultured at a concentration of 8 × 10^5^ cells/well for 72 h in the presence of either 20 μg/ml *L. major* FV1 or *L. mexicana* M379 FTAg, 2.5 μg/ml Concanavalin A (ConA) or media alone in a total volume of 200 μl/well. Culture supernatants were then removed and stored at -20 °C until analysis for IFNγ, IL-10, IL-4 and IL-13 levels using Duoset cytokine ELISA kits (R&D) according to the manufacturer’s instructions. The levels of antigen-specific IgG1 and IgG2c in plasma samples from mice were measured using Immunoglobulin Quantitation kits from Bethyl Labs according to the manufacturer’s instructions with minor modifications.

### Statistical analysis

Data were analysed with SPSS and GraphPad Prism 5 software, and figures generated using GraphPad Prism 5. As the data were found not to be normally distributed, groups were compared using the Mann-Whitney *U* test.

## Results

### TLR2 is important for controlling lesion development after infection with *L. major* promastigotes and *L. mexicana* promastigotes and amastigotes

**Fig. 1:**
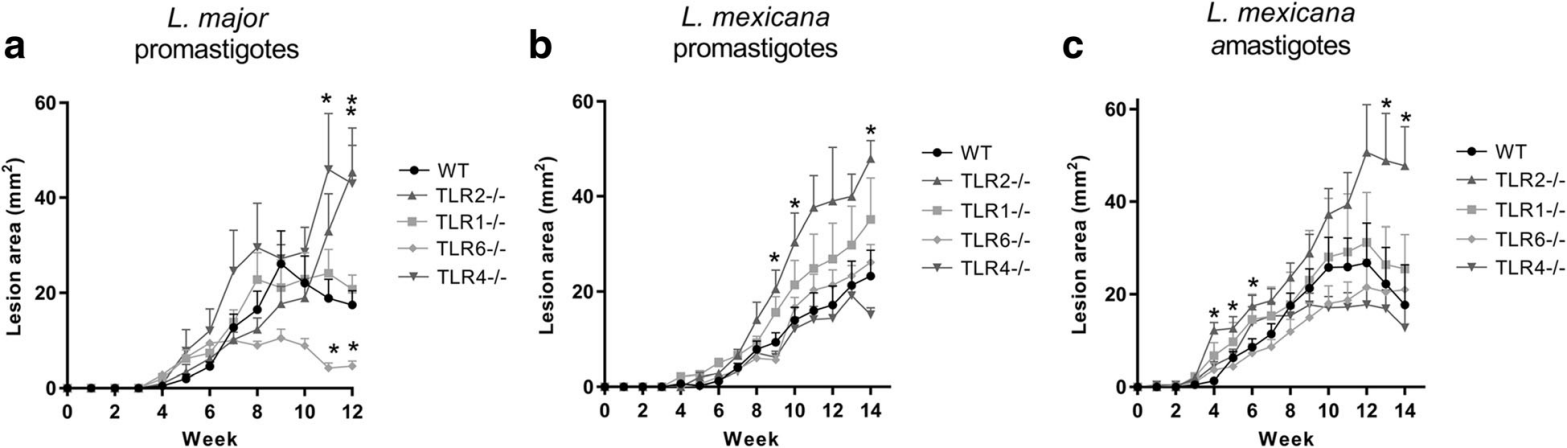
Lesion development in WT and TLR^−/−^ mice upon infection with *L. major* or *L. mexicana*. Either 10^5^ *L. major* promastigotes (**a**), or *L. mexicana* promastigotes (**b**) or amastigotes (**c**) were used to infect WT, TLR2^−/−^, TLR1^−/−^, TLR6^−/−^ and TLR4^−/−^ mice (*n* = 4–9) subcutaneously; mice were monitored every week for the appearance and size of lesions. The mean lesion size (mm^2^) + standard error for each genotype is shown at each weekly time point post-infection, for experiments ending between 12 and 14 weeks. For the *L. major* infection, the data presented is representative of two experiments. Knockout stains were compared to WT mice using the Mann-Whitney *U* test, where *P* < 0.05 was considered to indicate significant (*) differences

The role of TLR2, 1, 6 and 4 in lesion development in cutaneous leishmaniasis was explored by carrying out infection experiments using mice specifically deficient in these TLRs. The lesion development after infection of WT, TLR2^−/−^, TLR1^−/−^, TLR6^−/−^ and TLR4^−/−^ mice with either *L. major* and *L. mexicana* is presented in Fig. 1. After infection with either *L. major* promastigotes, *L. mexicana* promastigotes, of *L. mexicana* amastigotes, TLR2^−/−^ mice presented with significantly larger lesions than wild type (WT) C57BL/6 mice at one or more time points post-infection (p.i.). In all cases, the difference in lesion size was most pronounced at the peak of infection where lesion sizes were the greatest (Fig. 1). This suggests that TLR2 is important in controlling lesion development upon infection with both species. However, TLR2^−/−^ mice are still able to heal lesions in *L. major* infection (Additional file 1: Figure S1), and reduce lesion size in the later stages *L. mexicana* infection (Fig. 1c), suggesting that TLR2 is not essential for healing and control of parasite replication.

**Fig. 2:**
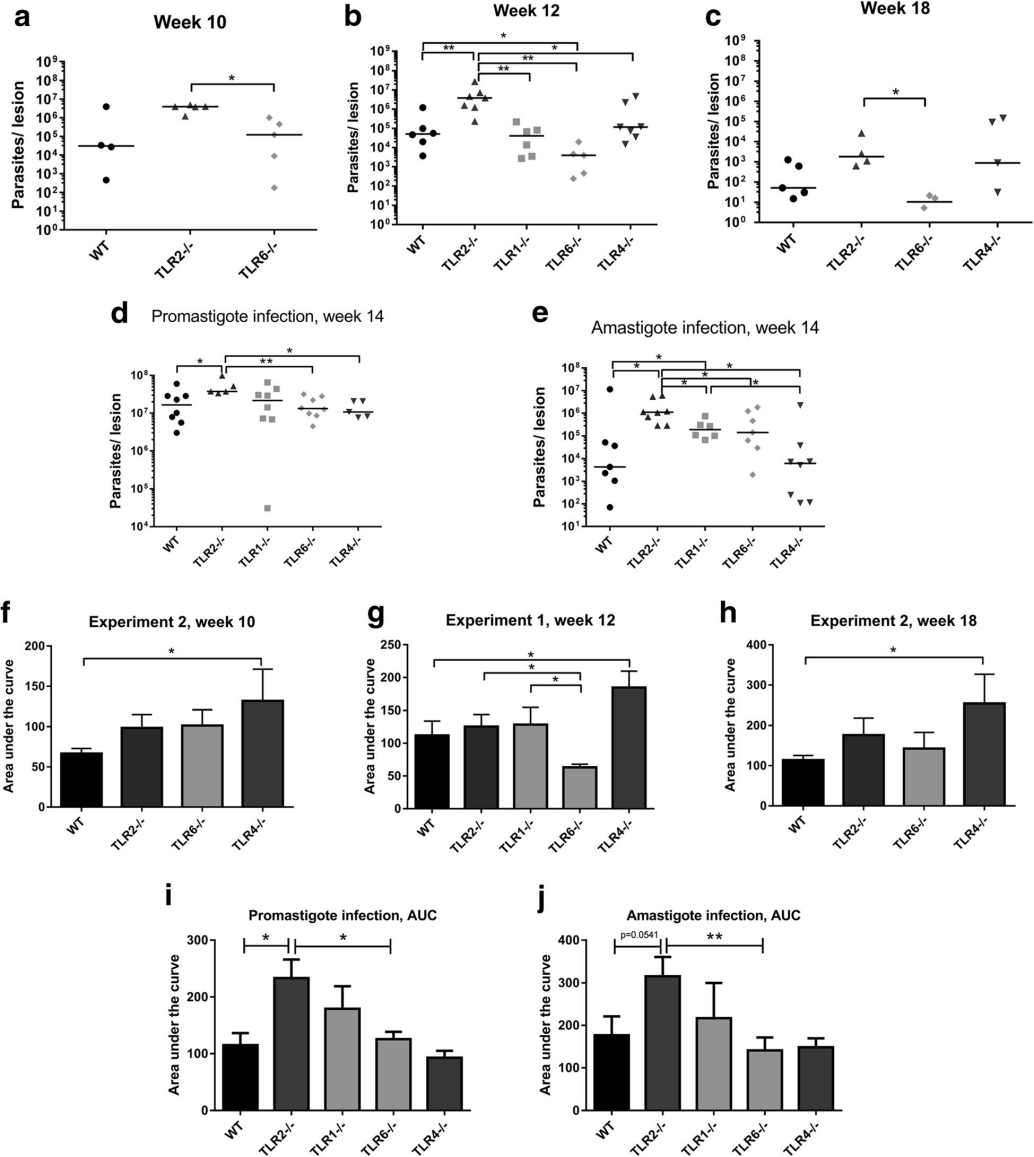
Parasite burden and area under the curve (AUC) analysis. Parasite burden and area under the curve data was calculated for WT and TLR^−/−^ mice infected with either *L. major* (**a**, **b**, **c**, **f**, **g**, **h**) or *L. mexicana* promastigotes (**d**, **i**) or amastigotes (**e**, **j**) at various time points post-infection. At the time points indicated, experiments were ended and the lesion tissue from infected mice was collected for quantification of the parasite burden using qPCR. The individual parasite burden levels are shown for *L. major* (**a**, **b**, **c**) as well as *L. mexicana* promastigote (**d**) and amastigote (**e**) infections, with the median average for each group shown. The lesion size data for each experiment can be summarized by calculating the AUC for each mouse at the end of the experiment; the data are presented in **f**, **g**, **h** for *L. major* and **i**, **j** for *L. mexicana*. The mean average for each group is represented by the bars, + SEM. Groups were compared using a Mann-Whitney *U* test where *P* < 0.05 was considered to indicate significant (*) differences

At necropsy the parasite burden in the lesions of infected mice was quantified by PCR amplification of kinetoplast minicircle (kmini) DNA from *Leishmania* extracted from the lesion tissue. These results indicate an increased susceptibility of TLR2^−/−^ mice to infection with both species, with higher average parasite burdens at all time points p.i. after lesion appearance, significantly so at 12 weeks p.i. (Mann-Whitney *U* = 1, *P* = 0.0023) with *L. major* (Fig. 2b), and 14 weeks p.i. after infection with both *L. mexicana* promastigotes (*U* = 4, *P* = 0.019) and amastigotes (*U* = 8, *P* = 0.021) (Fig. 2d, e). The area under the curve (AUC) analysis of lesion development also demonstrates elevated AUC values in *L. mexicana* infected TLR2^−/−^ mice when compared to WT mice after infection with either promastigote or amastigote parasites (Fig. 2i, j).

### TLR2 function during infection with either *L. major* or *L. mexicana* is not dependent on either known co-receptor, TLR1 or TLR6

TLR2 is known to recognise bacterial lipopeptides as a heterodimer with either TLR1 or TLR6. To explore whether the role of TLR2 in *L. major* and *L. mexicana* infections is dependent on either of these co-receptors, TLR1^−/−^ and TLR6^−/−^ mice were also infected with these parasites. TLR1^−/−^ mice showed no difference in lesion size at any time point after infection with *L. major* (Fig. 1a). However, TLR6^−/−^ mice infected with *L. major* did show significant differences in lesion size compared to WT mice, with healing occurring earlier resulting in significantly smaller lesions in the later stages of infection (weeks 11–12) (Week 11: *U* = 2, *P* = 0.0047; Week 12: *U* = 0, *P* = 0.0012) (Fig. 1a). In *L. mexicana* infection, none of the other knockout mice had significantly different lesion sizes compared to WT at any time point (Fig. 1b, c). However, TLR1^−/−^ mice did present with larger parasite burdens after infection with amastigotes at 4 weeks p.i. (*U* = 7, *P* = 0.046) (Fig. 2e). Nevertheless, the TLR2^−/−^ mice presented with significantly increased parasite burdens compared to TLR1^−/−^ mice in the same experiment (week 14: *U* = 8, *P* = 0.043), again showing that TLR2^−/−^ mice present with more severe disease overall than either TLR1^−/−^ or TLR6^−/−^ mice. Thus, TLR2 appears to function without a strict requirement for either known co-receptor, TLR1 or TLR6.

As the disease kinetics in TLR1^−/−^ or TLR6^−/−^ mice do not match that of TLR2^−/−^ mice, this strongly suggests that the role for TLR2 during infection does not require either TLR1 or TLR6. We attempted to generate mice deficient in both TLR1 and TLR6, but found after several attempts that no double knockout progeny were produced from breeding pairs of TLR1^−/−^ and TLR6^−/−^ mice.

### TLR4 plays a role in *L. major* infection but not in *L. mexicana* infection

In *L. major* infection, TLR4 also appears to play a role in controlling lesion development as has been shown previously (Fig. 1a) [12, 13], but is not crucial for healing as TLR4^−/−^ mice presenting with larger lesions were eventually resolved (Additional file 1: Figure S1). In contrast, TLR4 was not required for control of lesion development in *L. mexicana* infection (Fig. 1b, c). In fact, a trend towards smaller lesions and parasite burdens in TLR4^−/−^ mice was observed (although not significant), which is in contrast to that observed in *L. major* infection in the same mice. The difference between the role of TLR4 in infections with the two species is again shown by the average AUC values, as shown in Fig. 2f, g, h, with TLR4^−/−^ mice being the only mice with significantly elevated average AUC compared to WT in *L. major* infection (Exp. 1 week 12: *U* = 11, *P* = 0.05; Exp. 2 week 10: *U* = 2, *P* = 0.004; Exp. 2 week 18: *U* = 3, *P* = 0.05), yet no difference compared to WT in *L. mexicana* infection [promastigote infection (promas.): *U* = 19, *P* = 0.94; amastigote infection (amas.): *U* = 27, *P* = 0.96).

### The TLR2-mediated control of *L. mexicana* infection is not entirely dependent on activation by LPG

**Fig. 3:**
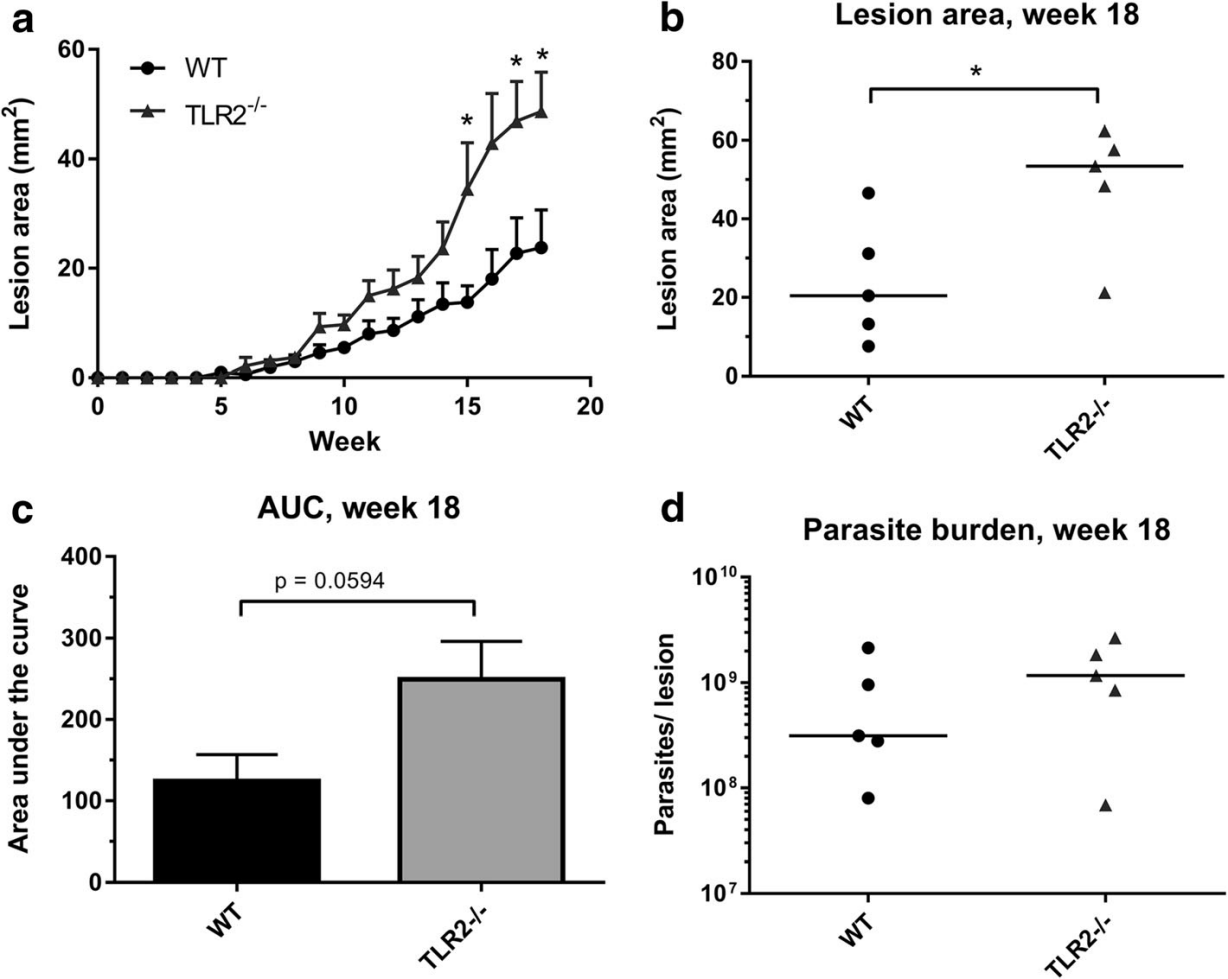
Infection of WT and TLR2^−/−^ mice with *L. mexicana LPG1* ^−/−^ promastigote parasites. WT and TLR2^−/−^ mice were infected with 10^5^ *L. mexicana LPG1* ^−/−^ parasites, and the disease was monitored by measuring the lesions every week for 18 weeks (*n* = 5). The average lesion size (mm^2^) + standard error (SEM) are displayed for both groups at all time points post-infection (**a**), and at the end of the experiment (week 18, **b**). The AUC was calculated for each mouse after the 18 weeks, the average is displayed (+SEM) in the bar chart in (**c**). The parasite burden in the lesion tissue was determined by qPCR, and individual burdens and median averages are displayed in (**d**). Groups were compared using a Mann-Whitney *U* test where *P* < 0.05 was considered to indicate significant (*) differences

As several studies have shown that *Leishmania* LPG is a ligand for TLR2 in vitro [10, 16], it was hypothesised that amastigotes, which lack expression of LPG, would give a different phenotype in TLR2^−/−^ infected mice (i.e. revert to the WT phenotype). However, the phenotypes of both amastigote and promastigote infections were strikingly similar, with TLR2^−/−^ mice developing larger lesions in the later stages of infection (promas. week 9: *U* = 6, *P* = 0.019. promas. week 10: *U* = 7, *P* = 0.028; promas. week 14: *U* = 4, *P* = 0.019; amas. week 13: *U* = 11, *P* = 0.05; amas. week 14: *U* = 9, *P* = 0.029) and presenting with higher parasite burdens than WT mice and other groups (promas. week 14: *U* = 4, *P* = 0.019; amas. week 14: *U* = 8, *P* = 0.021) (Fig. 1b, c). Next we carried out an infection experiment with *L. mexicana LPG1* ^−/−^ (GenBank: AJ271080.1) parasites, which specifically lack the LPG molecule on their surface. TLR2^−/−^ mice also developed larger lesions than WT mice when infected with *L. mexicana LPG1* ^−/−^ promastigote parasites (week 18: *U* = 2, *P* = 0.03) (Fig. 3a, b), suggesting that activation of TLR2 by LPG is not responsible for TLR2 mediated control of parasite replication in vivo. However, given that these *LPG1* ^*−/−*^ parasites are known to upregulate the expression of other phosphoglycan molecules [23], we cannot rule out that some of these molecules may be able to interact with TLR2.

### TLR2^−/−^ mice develop elevated Th2 cytokine responses during *L. mexicana* infection and elevated IgG1 production in *L. major* infection

**Fig. 4:**
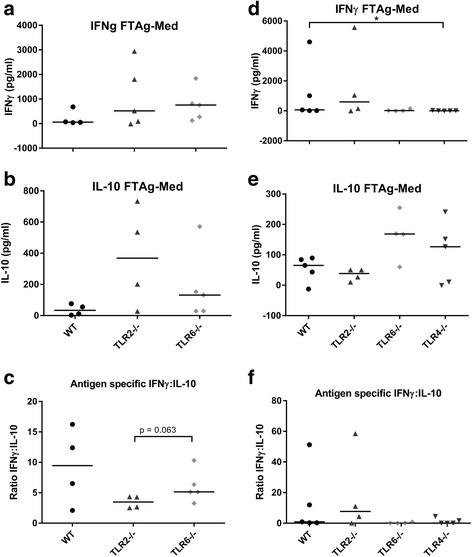
Antigen-specific cytokine production by DLNs in *L. major* infection in WT and TLR^−/−^ mice. WT, TLR2^−/−^ and TLR6^−/−^ mice were infected with *L. major* parasites and left to develop lesions for either 10 (**a**, **b**, **c**) or 18 weeks (**d**, **e**, **f**). For the long term experiment, TLR4^−/−^ mice were also included (**d**, **e**, **f**). At the end of the experiment, DLN were removed and the cells were re-stimulated for 72 h in vitro with the *Leishmania* antigen FTAg. The supernatants were collected and analysed for the presence of the cytokines IFNγ (**a**, **d**) and IL-10 (**b**, **e**) using ELISA. The quantities of cytokine produced in response to FTAg is shown for each individual, along with the median values. The ratio of IFNγ:IL-10 was also calculated for each individual and is displayed in **c** (week 10) and **f** (week 18). Groups were compared using a Mann-Whitney *U* test where *P* < 0.05 was considered to indicate significant (*) differences

**Fig. 5:**
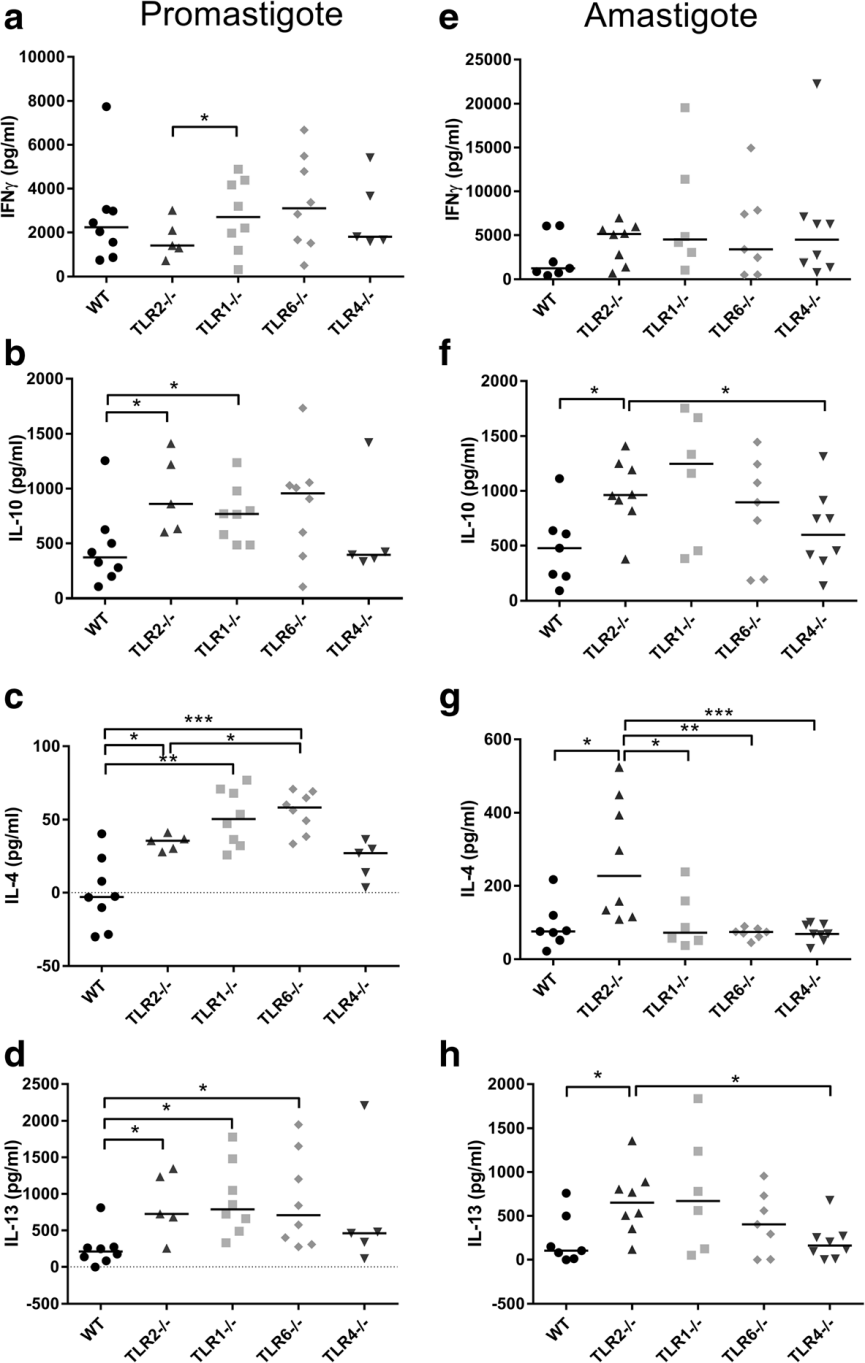
Antigen-specific cytokine production in *L. mexicana* infected WT and TLR−/− mice. WT, TLR2^−/−^, TLR1^−/−^, TLR6^−/−^ and TLR4^−/−^ mice were infected with *L. mexicana* parasites (promastigotes **a**, **b**, **c**, **d**, amastigotes **e**, **f**, **g**, **h**) and left to develop lesions for 14 weeks. At the end of the experiment, DLN were removed and the cells were re-stimulated for 72 h in vitro with the *Leishmania* antigen FTAg. The supernatants were collected and analysed for the presence of the cytokines IFNγ, IL-10, IL-4 and IL-13 using ELISA. The quantities of cytokine produced in response to FTAg are shown for each individual, along with the median values for each group. Groups were compared using a Mann-Whitney *U* test where *P* < 0.05 (*) and *P* < 0.01 (**) were considered to indicate significant differences

In order to explore whether the lack of specific TLRs results in a different type of adaptive immune response in infected mice, the draining lymph nodes (DLNs) of infected mice were isolated at the end of each experiment, and the cells re-stimulated ex vivo with the *Leishmania* antigen FTAg. In *L. major* infection, there were no striking differences in cytokine production shown by any of the groups which specifically lacked a TLR, when compared to WT mice (Fig. 4a, b, d, e). When the ratio of IFNγ:IL-10 production was calculated, an increased ratio was recorded in TLR6^−/−^ mice when compared to TLR2^−/−^ mice at week 10 p.i., which was almost significant (*U* = 2, *P* = 0.064), suggesting that TLR6^−/−^ mice have an enhanced ability to promote a Th1 immune response which is linked to enhanced resistance to infection (Fig. 4c). However, in *L. mexicana* infection, significantly elevated levels of IL-10, IL-4 and IL-13 were detected from DLN cells of mice lacking either TLR2 (IL-10 promas: *U* = 5, *P* = 0.030; IL-4 promas: *U* = 4, *P* = 0.019; IL-13 promas: *U* = 5, *P* = 0.030; IL-10 amas: *U* = 8, *P* = 0.021; IL-4 amas: *U* = 6, *P* = 0.0093; IL-13 amas: *U* = 7, *P* = 0.014), TLR1 (IL-10 promas: *U* = 13, *P* = 0.049; IL-4 promas: *U* = 3, *P* = 0.0011; IL-13 promas: *U* = 4, *P* = 0.0019) or TLR6 (IL-4 promas: *U* = 2, *P* = 0.0006; IL-13 promas: *U* = 4, *P* = 0.0019), stimulated with *L. mexicana* FTAg compared to WT (Fig. 5b, c, d, f, g, h), which indicates enhanced Th2 and regulatory responses in the local immune responses to the infection site in these mice.

**Fig. 6:**
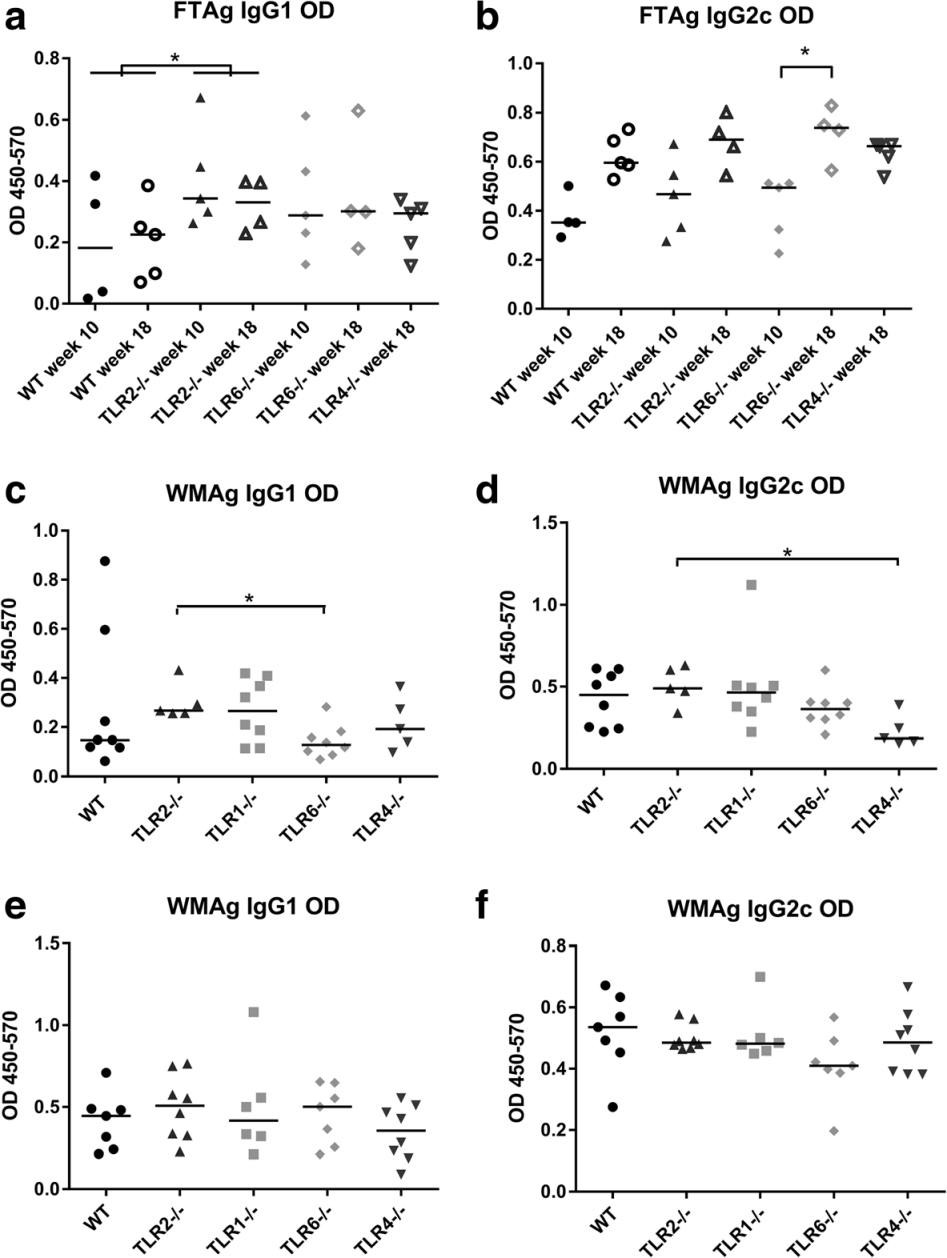
Levels of circulating antigen-specific IgG1 and IgG2c antibodies in infected WT and TLR^−/−^ mice. WT and TLR^−/−^ mice were infected with 10^5^ *L. major* promastigotes (**a**, **b**), *L. mexicana* promastigotes (**c**, **d**) or *L. mexicana* amastigotes (**e**, **f**). At the end of each experiment (week 10 or 18 for *L. major* infection, week 14 for *L. mexicana* infections), blood was collected from mice via cardiac puncture and the plasma was collected. Levels of antigen-specific antibodies were calculated using antibody ELISA with either *L. major* FTAg (**a**, **b**) or *L. mexicana* WMAg (**c**, **d**, **e**, **f**) as the capture antigen. Quantities are displayed as OD values for individual mice, as well as the median value for each group. Groups were compared using a Mann-Whitney *U* test *P* < 0.05 (*) and *P* < 0.01 (**) were considered to indicate significant differences

Isotype switching of antigen-specific antibodies by B cells is influenced by the production of different cytokines. The two major isotypes of circulating IgG are therefore biomarkers of the type of immune response, with IgG1 isotype indicating a Th2–biased response and IgG2a/c indicating a Th1 response in mice (due to a requirement of IL-4/IFNγ in IgG1/IgG2a-c isotype switching [24]). The levels of antigen-specific IgG1 and IgG2c antibodies present in the plasma of infected mice are displayed in Fig. 6. In *L. major* infected mice, the level of circulating antigen-specific IgG1 antibody did not change from week 10 to week 18 (WT: *U* = 9, *P* = 0.90; TLR2^−/−^: *U* = 7, *P* = 0.56; TLR6^−/−^: *U* = 8, *P* = 0.73) (Fig. 6a), whereas the median concentration of antigen-specific IgG2c increased in all groups from week 10 to week 18 (Fig. 6b), significantly so for TLR6^−/−^ mice (*U* = 0, *P* = 0.016). Thus the ratio of IgG1:IgG2c decreased in all groups from week 10 to week 18, indicating a shift towards a dominant Th1 immune response. The overall levels of antigen-specific IgG1 collected at both time points was significantly higher in the TLR2^−/−^ mice compared to WT mice (*U* = 17, *P* = 0.04), indicating an overall elevated Th2 response in these mice (Fig. 6a). In mice infected with *L. mexicana*, the antigen used was WMAg, an amastigote antigen preparation (and thus one which is reflective of the parasite exposed to the immune responses during chronic infection in vivo). Circulating antibody levels in mice infected with *L. mexicana* are shown in Fig. 6c–f (promastigotes and amastigotes respectively). Although the average levels of IgG1 were higher in infected TLR2^−/−^ mice compared to WT mice, no significant difference was seen (promas: *U* = 10, *P* = 0.17; amas: *U* = 19, *P* = 0.34), and no other TLR deficient group of mice had levels which differed from the WT mice. Of note, however, TLR6^−/−^ mice infected with *L. mexicana* promastigotes had significantly reduced levels of IgG1 when compared to TLR2^−/−^ mice (*U* = 3, *P* = 0.011) (Fig. 6c).

Experiments with both *L. major* and *L. mexicana* indicate that TLR2^−/−^ mice have elevated regulatory and/or Th2 response, as demonstrated either by elevated IL-10, IL-4 and IL-13 production (*L. mexicana*) or IgG1 circulating antibody (*L. major*). This is in contrast to the Th1 responses measured (IFNγ and IgG2c), where no difference between WT mice and mice deficient of TLRs was found.

## Discussion

The data presented here indicate a role for TLR2 in controlling infection with either *L. major* or *L. mexicana*, as mice lacking this receptor develop more severe disease and increased parasite burdens. In the *L. major* infection TLR2 was found to have a role in controlling development of lesions and in controlling parasite replication. Infection of TLR2^−/−^ mice with *L. mexicana* promastigotes yielded very similar results to that of *L. major*, with significantly increased lesions and parasite burdens. This is the first study, to our knowledge, to attribute a clear role for TLR2 in the control of infection in cutaneous leishmaniasis. In addition we confirm a role for TLR4 in *L. major* infection of mice, which mount a healing phenotype (C57BL/6 background), as previously reported by others [12, 13], although the TLR4-dependent activity does not extend to *L. mexicana* infection, in contrast to our observations with TLR2.

Interestingly, Murray et al. [25] showed that when *L. donovani*, which causes visceral leishmaniasis, is used to infect the same WT, TLR2^−/−^ and TLR4^−/−^ mice as used in our study, a contrasting role for the two TLRs is found whereby a lack of TLR2 leads to enhanced and sustained reduction in parasite replication in the liver, whilst a lack of TLR4 leads to increased parasitaemia at the peak of infection. The contrasting role for TLR2 reported between our study and theirs could be explained by a differing role for TLRs in distinct sites of parasite replication (skin versus liver), route of infection (subcutaneous versus intravenous) and/or a different role for TLRs in the immune response to different species of *Leishmania*. Indeed, our results show that while the role for TLR2 is similar for *L. mexicana* and *L. major* infection, TLR4 plays a role only in infection with *L. major*. Vargas-Inchaustegui et al. [26] demonstrated that TLR2^−/−^ mice presented with reduced lesions sizes at the peak of infection (weeks 3–5) when compared to WT mice, after infected with *L. braziliensis*, a new world species which results in muco-cutaneous disease in humans. However, no difference in parasite load was found in this study, indicating a role for TLR2 in lesion development/healing in the setting of *L. braziliensis*, rather than in increasing parasite load [26]. When combined, the in vivo studies exploring the role of TLR2 in *Leishmania* infections by us and others illustrate that the influence of TLR2 in vivo is complex and can exert profoundly different species-(or disease type-)-dependent outcomes.

Studies utilising TLR9^−/−^ mice infected with *L. major* showed similar disease kinetics to those reported here with TLR2^−/−^ and TLR4^−/−^ mice, with increased lesion sizes and parasite burdens during the acute phase of infection with eventual control of the disease [14, 15]. A more recent study showed that the three nucleic acid sensing TLRs, TLR3, 7 and 9, are crucial for a protective response against *L. major* infection, as mice which lacked all of these functional TLRs (i.e. TLR3/TLR7/TLR9^−/−^ or UNC93B1^−/−^ mice) were highly susceptible to infection [27]. Furthermore, Schamber-Reis et al. [27] showed that a combination of all three of these TLRs is important, as neither the single or double knockout equivalent mice developed the same susceptible phenotype. Therefore, it appears that multiple TLRs are involved in the detection of *Leishmania* parasites and promotion of healing responses in cutaneous leishmaniasis caused by *L. major*. In the *L. mexicana* infection model, we were able to explore whether the mechanism of TLR2 mediated control was due to activation by LPG by using parasites lacking LPG either with amastigote stages, which naturally lack LPG, or with genetically modified promastigotes which lack the expression of a full LPG molecule (*LPG1* ^−/−^). As TLR2^−/−^ mice developed more severe disease when infected with these LPG-lacking parasites as well as WT promastigotes, when can infer that the activation of TLR2 by LPG is not the sole mechanism of TLR2 mediated control in this species.

Although the *LPG1* ^−/−^ parasites used in this experiment lack a full LPG molecule, they retain the ability to synthesize the membrane anchor of LPG, which includes the acyl group that was found to be crucial for TLR2 activation [10]. It is not known whether the anchor of LPG is still expressed in high levels in the promastigotes of *LPG1* ^−/−^ parasites. Furthermore, it has been suggested that the phosphoglycan chain, which is absent in the *LPG1* ^−/−^ parasites, has an important role in the ability to activate TLR2, as shown by other studies comparing LPG isolated from different *Leishmania* species [28]. Osanya et al. [29] showed that synthetically produced tri-mannose molecules based on the cap of LPG (and ManLAM of *M. tuberculosis*), when coated onto the surface of synthetic beads, were able to signal through TLR2 and mannose receptor and enhance protective Th1 responses when administered with *L. major* parasites in vivo. However, the aforementioned study is the first to attribute the TLR2 activating ability of LPG to the mannose cap, and is in contrast to most studies using purified LPG which attribute the ability to activate TLR2 to the lipid moiety of the GPI anchor [10, 16], and indeed to other studies of TLR2 ligands which have determined the crucial acyl group required for efficient TLR2 activation [30–33]. To determine the precise mechanism of TLR2 activation by LPG and/or other parasite derived glycosylated molecules, it would be useful to determine the crystal structure of the ligand-receptor complex, as has been achieved for other complexes such as LPS-TLR4-MD2, Pam2-TLR2/6, Pam3-TLR2/1 and dsRNA-TLR3 [34, 35].

Infections with mice lacking either TLR1 or TLR6, the known co-receptors for TLR2, did not present with the same disease phenotype as TLR2^−/−^ mice, suggesting that neither is crucial for the TLR2-mediated control of infection with *L. major* or *L. mexicana*. The absence of an apparent role for TLR1 or TLR6 in the TLR2-mediated control of *L. major* and *L. mexicana* could implicate a ligand for TLR2 which has an alternative interaction with the receptor to that known for bacterial acylated TLR2 ligands, where the ligand-receptor complex has been elucidated in more detail. In these cases, the heterodimerisation of TLR2 with either co-receptor determines the specificity of the receptor for its ligand, with TLR2/6 recognising triacylated lipoproteins/lipopeptides [30] and TLR2/1 recognising diacylated lipoproteins/lipopeptides [31, 36]. Whilst increased resistance to *L. major* by mice lacking TLR6 was observed, TLR6^−/−^ mice did not have any reduced disease severity or parasite burdens upon infection with *L. mexicana* in this study. This may suggest that TLR6 acts to exacerbate infection with *L. major*, but not *L. mexicana,* or may perhaps be a reflection of the more chronic nature of *L. mexicana* infection, and in the reduced Th1 response involvement when compared to *L. major*. Due to our inability to exclude redundancy between TLR1 and TLR6 in these infection models, we are unable to rule out the possibility that TLR2 may utilise either TLR1 or TLR6 co-receptor involvement in these settings. Given that generating double knockout TLR1/6^−/−^ mice was not possible, further studies utilising other gene knockdown approaches (such as small interference (si)RNA) would be required to fully ascertain whether either co-receptor is involved in the in vivo TLR2-mediated role we have demonstrated in this study. In addition, siRNA techniques would allow us to ascertain the role for different receptors and co-receptors in the detection and response to *Leishmania* parasites by different immune cell subsets in vitro*.*

Kropf et al. [12, 13] found similar results in terms of kinetics of infection in the absence of TLR4 when using *L. major* LV39 in a similar infection model; lesions in C57BL/10ScN mice which lack a functional TLR4 gene had larger lesions just after the acute phase of infection (day 53), and higher parasite burdens at several time points (early and late stages of infection), when compared to their WT counterparts (C57BL/10ScSn). The 10ScN mice were found to produce elevated Th1 and Th2 cytokine responses to *L. major* in re-stimulated DLN (compared to ScSn or WT) including both IFNγ and IL-10, which was not repeated in this study as only elevated IFNγ was observed, although the time points post-infection at which DLN were taken was very different: week 4 p.i. in [13] and week 18 p.i. in this study. An additional finding was that macrophages from mice lacking TLR4 were found to produce more arginase in response to *L. major* infection when compared to TLR4-competent macrophages, suggesting that TLR4 plays a role in preventing alternative activation of macrophages during infection independently of the adaptive immune response [13]. A role for neutrophil elastase (NE) in the activation of *L. major* infected macrophages to kill via TLR4 was provided in a study by Ribeiro-Gomes et al. [37], where it was demonstrated that neutrophils were able to induce intracellular killing in a TNFα and TLR4 dependent manner, and NE was responsible for this effect. Thus a host-derived TLR4 ligand, or damage-associated molecular pattern (DAMP), is potentially linked with the role of TLR4 in *L. major* control.

The dynamics of the role of TLR2 on *Leishmania* infection are such that the effect of TLR2 activation on disease severity manifests several weeks after initial infection, and appears to function to promote an effective healing response by reducing detrimental immune responses such as Th2 cytokine production and elevated IgG1 levels. Activation of TLR2 by co-injection of a synthetic TLR2 ligand at the time of infection, has been shown to promote the production of protective cytokines in the context of *L. major* infection before [38]. In our experiments, we did not see changes in the levels of IFNγ production in infected mice lacking TLR2, but we did see increased IL-10, IL-4 and IL-13 levels in *L. mexicana* infections. The lack of differences in IFNγ observed in our experiments may reflect the relatively late time points post-infection at which we measured the cytokine response. Several studies have linked *Leishmania*-specific IgG [39, 40], and specifically IgG1 antibody isotypes [20] to severity of infection with *Leishmania* spp. It is believed that during infection, amastigotes are able to infect new macrophages via IgG antibody receptors (FcγRs), which results in production of IL-10, thereby regulating protective responses at the site of infection (e.g. classical macrophage activation) and allowing further parasite replication [41].

A lack of TLR2 does not prevent the eventual resolution of the infection, suggesting that other immune components are important for parasite clearance. Nevertheless, the results we have presented improve our understanding of how *Leishmania* parasites interact with TLRs during infection in vivo, and how this interaction impacts on immune responses and disease outcome. In these models, it was found that TLR9 in DCs is activated by *L. major* DNA and this activation promotes priming of a protective Th1 response via production of IL-12, activation of NK cells and IFNγ production, which all act to promote parasite killing by NO production by macrophages and to suppress non-protective Th2 responses [14, 15]. Nevertheless, infected TLR9^−/−^ mice were able to mount an appropriate Th1 response and heal their lesions, and the deficiency appeared to be a delayed ability to control non-protective Th2 responses. Thus, neither TLR2 nor TLR4 nor TLR9 is solely responsible for the important role of MyD88 in mounting a protective response to *L. major*, where mice deficient in MyD88 develop uncontrollable disease and insufficient Th1 or ineffective responses [9, 42]. It is known that activation of more than one TLR can have either a complimentary, synergistic or antagonistic effect on innate immune responses (and subsequent adaptive immune responses) [43], and it may well be that it is a combination of TLRs that cooperate synergistically, all via MyD88 signalling, to orchestrate protective responses. Such a phenomenon appears to be the case in infection with a related intracellular protozoan parasite, *T. cruzi,* where mice deficient in both TLR2 and TLR9 were found to be more susceptible than mice deficient in either one receptor, and the TLR2^−/−^TLR9^−/−^ dual deficient mice had levels of susceptibility comparable to that of mice deficient in MyD88 [44].

Although the protective influence of TLR2 was consistently observed in our experiments for both *L. major* and *L. mexicana,* other studies using the species *L. braziliensis* and *L. donovani*, have demonstrated an exacerbatory role for TLR2 during infection [25, 26, 45], illustrating the influence of TLR2 is complex and can exert profoundly different species-dependent outcomes. Our findings further suggest that the ligand for the TLR2-mediated effects in vivo is not, at least exclusively, LPG, and that if the ligand in question is parasite derived, it is expressed by amastigotes in *L. mexicana* infection. Whilst others have shown activation of TLR2 by LPG preparations in vitro resulting in inflammatory responses [10, 16, 18], it is important to note that this is in contrast to many of the known functions of LPG in vivo*,* which are related to the down regulation of inflammatory responses [46–49], so it would be paradoxical for LPG to also promote protective immune responses in the context of an in vivo infection. Further research is needed to understand which host cells are involved in the TLR2 interaction with *Leishmania*, and to determine whether the activator of TLR2 is in fact derived from the parasite, or an alternative source, such as other microbes present at the infection site, or host damage-associated molecular patterns (DAMPs). An interesting area of research is the impact of resident skin microflora at the lesion site [50], which play an important role in lesion development and immunity to *L. major*. It would be interesting to further explore the role of the skin microbiota in relation to TLR2 activation in cutaneous leishmaniasis in mice.

## Conclusions

In summary, a role for TLR2 in controlling cutaneous leishmaniasis disease severity has been demonstrated in vivo*.* The absence of this phenotype in either TLR1^−/−^ or TLR6^−/−^ mice suggests that TLR2 does not have a specific requirement for either known co-receptor during *Leishmania* infection. Furthermore, experiments using *L. mexicana* parasites, which lack LPG, indicate that LPG is not required for the observed TLR2 mediated effects in vivo.

## Additional file

Additional file 1: Figure S1. Lesion development in WT and TLR^−/−^ mice upon infection with *L. major* after 18 weeks. WT, TLR2^−/−^, TLR1^−/−^, TLR6^−/−^ and TLR4^−/−^ mice (*n* = 4–5) were infected with 10^5^ *L. major* promastigotes subcutaneously. Mice were monitored every week for the appearance and size of lesions. The mean lesion size (mm^2^) + SEM for each genotype is shown at each weekly time point post-infection up to the end of the experiment at 18 weeks. Knockout stains were compared to WT mice using the Mann-Whitney *U* test, where *P* < 0.05 was considered to indicate significant (*) differences. (TIF 28 kb)

## Abbreviations

DLN: Draining lymph node
FTAg: Freeze-thaw antigen
IFNγ: Interferon gamma
IgG: Immunoglobulin G
IL: Interleukin
IL-1R: Interleukin 1 receptor
LPG: Lipophosphoglycan
MyD88: Myeloid differentiation primary response gene 88
NO: Nitric oxide
SOCS: Suppressor of cytokine signalling
TLR: Toll-like receptor
TNFα: Tumour necrosis factor alpha
WT: Wild-type
